# Molecular diversity of exotic durian (*Durio* spp.) germplasm: a case study of Kalimantan, Indonesia

**DOI:** 10.1186/s43141-022-00321-8

**Published:** 2022-03-01

**Authors:** Dindin Hidayatul Mursyidin, Muhammad Irfan Makruf, Badruzsaufari, Aidi Noor

**Affiliations:** 1 Laboratory of Genetics and Molecular Biology, Faculty of Mathematics and Natural Sciences, University of Lambung Mangkurat, Jl. A. Yani Km. 36, Banjarbaru, South Kalimantan 70714 Indonesia; 2 Assessment Institute of Agricultural Technology, Jl. Panglima Batur Barat No. 4, Banjarbaru, South Kalimantan 70714 Indonesia

**Keywords:** DNA barcoding, Genetic diversity, Malvaceae, Phylogenetic relationship, Plant breeding

## Abstract

**Background:**

Durian of Indonesia, specifically *Durio zibethinus*, is a potential agricultural commodity for domestic and international markets. However, its quality is still less competitive or significantly lower to fulfill the export market, compared to a similar one from other countries. This study aimed to determine and analyze the genetic diversity and relationship of the exotic durian (*Durio* spp.) germplasm originally from Kalimantan, Indonesia, using the *rbc*L marker.

**Results:**

Based on this marker, the durian germplasm has a low genetic diversity (π%=0.24). It may strongly correspond with the variability sites or mutation present in the region. In this case, the *rbc*L region of the durian germplasm has generated 23 variable sites with a transition/transversion (Ti/Tv) bias value of 1.00. However, following the phylogenetic and principal component analyses, this germplasm is separated into four main clades and six groups, respectively. In this case, *D. zibethinus* was very closely related to *D. exleyanus*. Meanwhile, *D. lowianus* and *D. excelsus were* the farthest. In further analysis, 29 durians were very closely related, and the farthest was shown by *Durian Burung* (*D. acutifolius*) and *Kalih Haliyang* (*D. kutejensis*) as well as *Pampaken Burung Kecil* (*D. kutejensis*) and *Durian Burung* (*D. acutifolius*) with a divergence coefficient of 0.011. The Pearson correlation analysis confirms that 20 pairs of individual durians have a strong relation, shown by, e.g., *Maharawin Hamak* and *Durian Burung* as well as *Mantuala Batu Hayam* and *Durian Burung Besar.*

**Conclusion:**

While the durian has a low genetic diversity, the phylogenetic analyses revealed that this germplasm originally from Kalimantan, Indonesia, shows unique relationships. These findings may provide a beneficial task in supporting the durian genetic conservation and breeding practices in the future, locally and globally.

## Background

Durian, specifically *Durio zibethinus*, is a potentially valuable agricultural commodity for domestic and international markets [1]. Even it is a prospective export commodity today. For example, Indonesia, one of the world’s biggest durian producers, was able to sell this fruit to numerous other countries, including several Middle Eastern countries with a total value of 232,000 USD in 2020 [2]. Similarly, this country has generated over 1.19 million metric tons of durian in the same year [3]. However, compared to a similar commodity from two adjacent nations, i.e., Thailand and Malaysia, the quality of Indonesian durians is still less competitive (significantly lower) to fulfill the export market [4]. As a result, various strategic plans must be included in the breeding program to improve the quality of durian fruit, particularly.

Germplasm collection is a vital component of plant breeding program success or the development of new superior cultivars [5]. Around 18 of the world’s 27 durian species, including their wild relatives, have been discovered in Indonesia. There are even 16 species that are considered endemic, and the Kalimantan is the Indonesian island with the highest durian genetic diversity [6]. According to Uji [7], the nine edible durian species present in this region with delicious flesh taste and unique fruit characteristics, including *D. dulcis*, *D. excelsus*, *D. grandiflorus*, *D. graveolens*, *D. kutejensis*, *D. lowianus*, *D. oxleyanus*, *D. testudinarum*, and *D. zibethinus*. In addition, these durians have other advantages, such as being resistant to diseases, especially patch canker, and having a high tolerance for environmental challenges, such as acid soil [6]. As a result, the germplasm is useable as a parent in a breeding effort.

Germplasm characterization is also crucial to aiding the durian plant breeding initiative or generating new superior cultivars [5]. However, durian germplasm and its relatives have only been characterized using morphological markers so far [1]. While these markers have advantages, they are frequently multigenic and heavily impacted by environmental factors [8]. Furthermore, morphological ones are inefficient since they can only be applied to mature plants, wait for the generative phase (flowers), and are time-consuming to apply [1]. The genetic diversity and relationships of durians have already been studied using various molecular markers, including RAPD [1, 9, 10], SSR, and ISSR [11, 12]. However, because these markers are highly subjective, the study is less precise [13]. According to Wu et al. [14], these markers are also poor consistency, limited repeatability, or complicated operation limit their effectiveness.

This study aimed to determine and analyze the genetic diversity and relationship of the exotic durian (*Durio* spp.) germplasm originally from Kalimantan, Indonesia, using the *rbc*L marker. Following Moura et al. [15], this marker shows high universality and amplification success. Also, this marker has generated a relatively high-quality output and unbias sequence data [13, 16]. So it is useful for discriminating Angiosperms or germplasm with close genetic relationships [17]. In partial, this marker has been applied for various plant germplasm, such as *Oryza sativa* [18], *Amorphophallus* [19], *Flacourtia inermis* [20], and *Ilex* [21]. As a result, the findings of this study can be applied to help future durian germplasm breeding initiatives, both locally and globally.

## Methods

### Plant samples

**Fig. 1:**
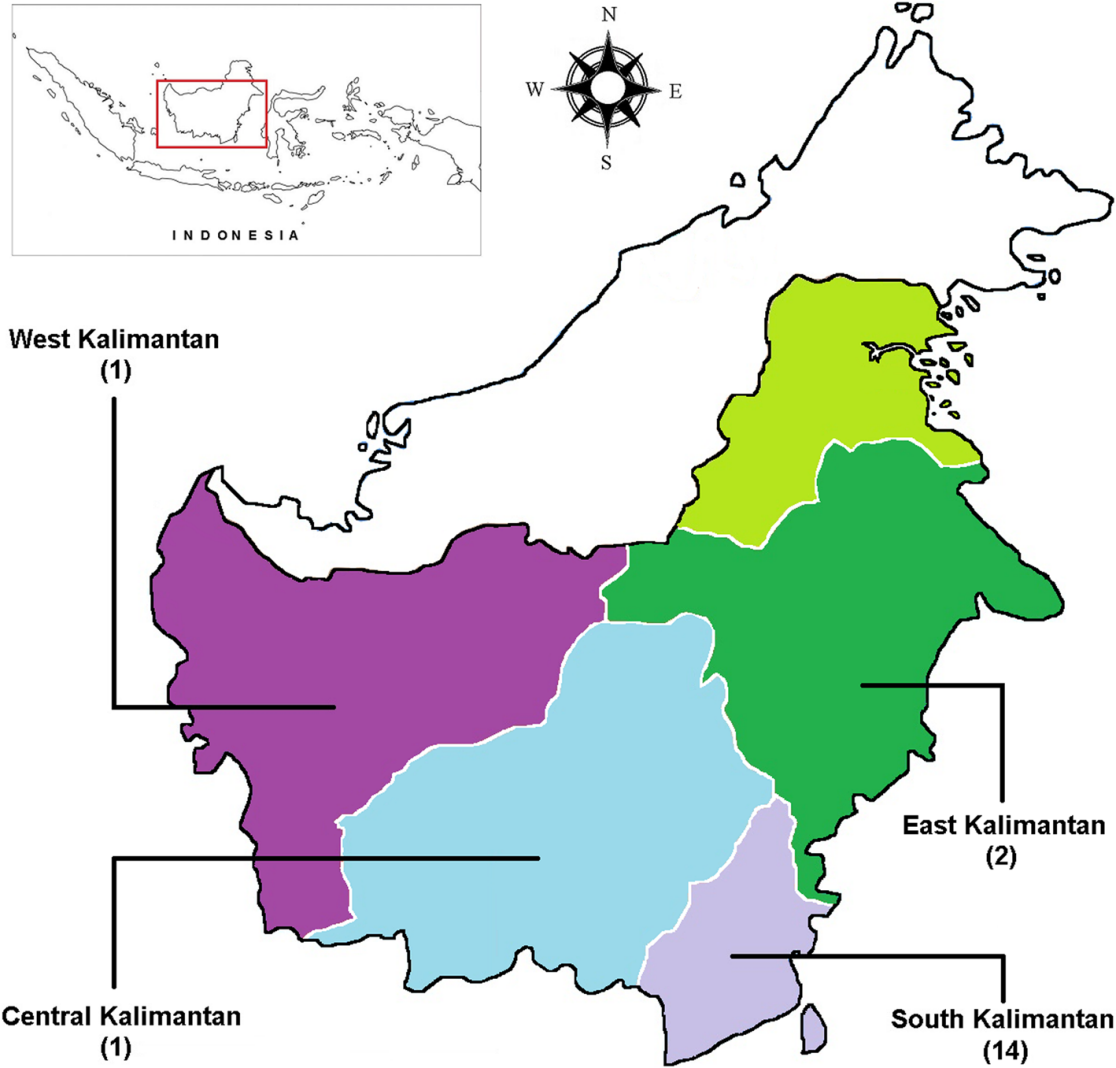
Map of Kalimantan, Indonesia, showing sampling locations where eighteen durians were collected and used in this study. The list of durian samples and their complete origin are shown in Table 1

In this study, we have used a total of eighteen samples of durian (*Durio* spp.) germplasm, excluding an outgroup (Table 1). Most of the durian samples were collected from South Kalimantan, Indonesia, using a purposive sampling method (Fig. 1). Meanwhile, an outgroup (*Bombax ceiba*) was obtained from the GenBank database.

**Table 1:** List of exotic durian (*Durio* spp.) germplasm used in this study, including their origin, GenBank accession number, and *rbc*L sequence length

Local name	Code	Species	Origin	Ordinate	Acc. Num.	***rbc***L (bp)
*Durian Likol*	1	*D. zibethinus*	Tabalong, South Kalimantan	1° 37′ 04.84″S; 115° 31′ 14.65″E	MZ479693	566
*Durian Sahang*	2	*D. zibethinus*	Tabalong, South Kalimantan	1° 51′ 51.49″S; 115° 34′ 05.19″E	MZ479694	571
*Durian Si Japang*	3	*D. zibethinus*	Banjar, South Kalimantan	3° 29′ 11.54″S; 114° 58′ 31.50″E	MZ479695	529
*Kalih Haliyang*	4	*D. kutejensis*	Balangan, South Kalimantan	2° 19′ 33.75″S; 115° 36′ 55.70″E	MZ479679	571
*Kamundai*	5	*D. kutejensis*	Tabalong, South Kalimantan	1° 51′ 51.49″S; 115° 34′ 05.19″E	MZ479691	564
*Lai Lidung*	6	*D. kutejensis*	Kutai, East Kalimantan	0° 08′ 01.20″S; 116° 36′ 29.40″E	MZ479692	566
*Pampaken*	7	*D. kutejensis*	Tabalong, South Kalimantan	1° 51′ 51.49″S; 115° 34′ 05.19″E	MZ479690	568
*Pampaken Burung Kecil*	8	*D. kutejensis*	South Hulu Sungai, South Kalimantan	2° 43′ 18.34″S; 115° 12′ 02.78″E	MZ479683	568
*Durian Daun*	9	*D. lowianus*	South Hulu Sungai, South Kalimantan	2° 43′ 18.34″S; 115° 12′ 02.78″E	MZ479686	564
*Durian Malutu*	10	*D. lowianus*	South Hulu Sungai, South Kalimantan	2° 52′ 43.50″S; 115° 16′ 40.19″E	MZ479684	568
*Lahung Alang*	11	*D. lowianus*	Balangan, South Kalimantan	2° 19′ 33.75″S; 115° 36′ 55.70″E	MZ479688	568
*Durian Burung Besar*	12	*D. excelsus*	Balangan, South Kalimantan	2° 19′ 33.75″S; 115° 36′ 55.70″E	MZ479680	578
*Mantuala Batu Hayam*	13	*D. excelsus*	Central Hulu Sungai, South Kalimantan	2° 40′ 11.84″S; 115° 29′ 49.37″E	MZ479682	570
*Maharawin Hamak*	14	*D. oxleyanus*	Banjar, South Kalimantan	2° 45′ 33.03″S; 115° 20′ 56.21″E	MZ479681	527
*Karantungan Besar*	15	*D. oxleyanus*	Katingan, Central Kalimantan	0° 58′ 33.02″S; 112° 48′ 37.98″E	MZ479689	565
*Durian Burung*	16	*D. acutifolius*	Balangan, South Kalimantan	2° 19′ 33.75″S; 115° 36′ 55.70″E	MZ479678	527
*Lahung*	17	*D. dulcis*	Balangan, South Kalimantan	2° 19′ 33.75″S; 115° 36′ 55.70″E	MZ479687	585
*Durian Kura-Kura*	18	*D. testudinarium*	Sekadau, West Kalimantan	0° 18′ 54.35″S; 110° 51′ 19.05″E	MZ479685	568
*Indian Kapok*^a^	19	*Bombax ceiba*	Kerala, India	-	KY556637	747

^a^An outgroup, obtained from GenBank database

### DNA assay

The DNAs were extracted from the durian leaves using a combined commercial kit from Molecular Research Center Inc., USA (DNAZol@Direct) and Geneaid Biotech Ltd., Taiwan (GP100). The genetic materials were then quantified using a UV-VIS spectrophotometer (NanoVue, GE Healthcare, UK) and amplified by a pair of *rbc*L primers, namely *rbc*L-F (5′-ATGTCACCACAAACAGAGACTAAAGC-3′) and *rbc*L-R (5′-GTAAAATCAAGTCCACC RCG-3′) [22]. Amplification was employed in the total volume reaction of 25 μL, consisting of 2 μL DNA template (20 ng), 1 μL for each primer (0.2 μmol), and 22 μL of MyTaq HS Red Mix (Bioline, UK). This reaction was setting with the following conditions: initial denaturation at 94 °C for 5 min; followed by 35 cycles of denaturation at 94 °C for 30 s, annealing at 48 °C for 30 s, and extension at 72 °C for 45 s; and a final extension at 72 °C for 7 min [18]. The amplicon (DNA product) was then separated on 2% agarose gel electrophoresis, including a 1X TBE buffer solution and DNA stain (GelRed, Biotium Inc., USA), and observed on UV transilluminator. The DNA product was then purified and sequenced by 1st Base Ltd., Malaysia, using the Sanger method, bi-directionally. All sequence (*rbc*L) targets were deposited in GenBank with accession numbers of MZ479678–MZ479695 (Table 1).

### Data analysis

The *rbc*L sequences of durian were refined manually to a reconstructed consensus using the MEGA-X software [23]. These sequences were then aligned using Clustal-Omega [24] and MultAlin [25]. The genetic diversity of this germplasm was determined using the nucleotide diversity index (π%) with the following categories: 0.1–0.4 is low, 0.5–0.7 is moderate, and 0.8–2.00 is high [26]. The phylogenetic analysis was done by maximum likelihood (ML) and neighbor-joining (NJ) methods, with the assistance of MEGA-X [23]. The internal node of each phylogenetic tree was evaluated by the bootstrap method (1000 replicates) [27]. The genetic relationship was also determined using the principal component analysis (PCA) by the MVSP ver. 3.1 [28]. The AMOVA and evolutionary divergence among sequences were analyzed using the Kimura 2-parameter model [29]. In addition, the F-statistics at the molecular level were calculated among species (populations), and their significance was also tested by a permutation procedure, using 2000 permutations. These analyses were conducted by Arlequin [30]. The Pearson correlation (*r*) analysis, by the criteria of weak (*r* ≤ 0.35), moderate (*r* = 0.36–0.67), and strong (*r* > 0.68), was finally applied to confirm the differences in genetic structure between durian samples [31].

## Results

### The *rbcL* region of durians and its genetic diversity

**Fig. 2:**
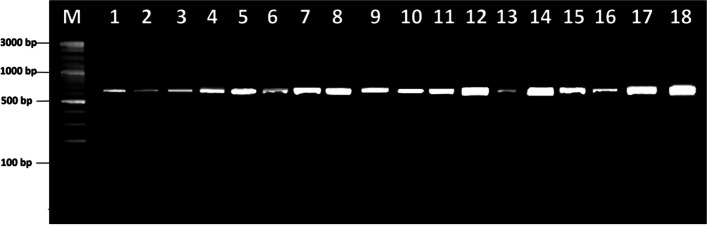
Electrophoresis profile showing PCR products of exotic durian germplasm generated by the *rbc*L marker. The name of each durian sample is shown in Table 1

**Fig. 3:**
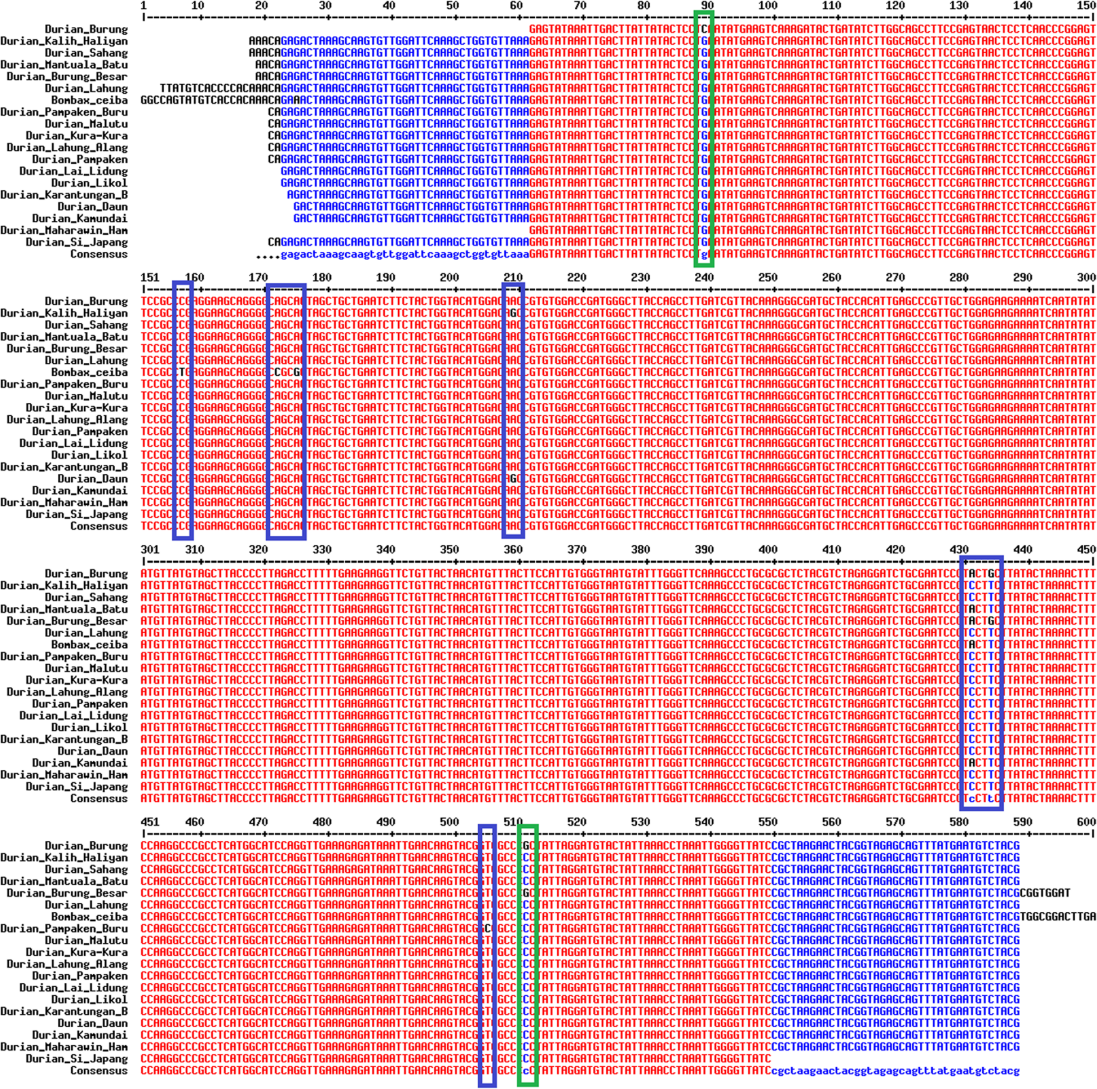
Multiple sequence alignment of *rbc*L of exotic durian (*Durio* spp.) germplasm, showing several mutational events, i.e., transition (green closed rectangle) and transversion (blue closed rectangle)

The durian *rbc*L region was successfully amplified. The amplification results show that this region has a size of approximately 650 bp (Fig. 2). After sequencing, each durian (*Durio* spp.) sample had a different *rbc*L sequence length, ranging from 527–578 bp (Table 2). The multiple sequence alignment is presented in Fig. 3. Based on Table 2, this region has 44.39% of GC content, 23 variable sites, five Parsimony informative sites, six singleton sites, and 0.51 transition/transversion bias values. Table 3 shows detailed information on the Parsimony informative site’s position on the *rbc*L region of the durian germplasm. In this case, only two mutations were present in this region, namely substitution-transition and substitution-transversion (Table 3) or no indels therein (Table 2). Furthermore, following this region, the durian germplasm has a nucleotide diversity (π%) of 0.24, with the AMOVA shown in Table 4. Following Table 4, at inter-species, the durian germplasm has a lower variation (5.62%) than the intra-species level (94.38%).

**Table 2:** Genetic information of *rbc*L sequences of durian (*Durio* spp.) germplasm

Parameter	***rbc***L
Range of sequence length (bp)	527–578
GC content (%)	44.30
Number of variable sites (*S*)	23
Number of parsimony informative sites	5
Number of singleton sites	6
Number of indels	0
Transition/transversion bias value (*R*)	1.00
Nucleotide diversity (π%)	0.24

**Table 3:** Parsimony informative sites on the *rbc*L sequences of durian (*Durio* spp.) germplasm

Local name	Species	Nucleotide position				
		209^**b**^	431^**b**^	434^**b**^	511^**a**^	748^**a**^
*Durian Likol*	*D. zibethinus*	.	C	T	C	.
*Durian Sahang*	*D. zibethinus*	.	C	T	C	G
*Durian Si Japang*	*D. zibethinus*	.	C	T	C	G
*Kalih Haliyang*	*D. kutejensis*	G	C	T	C	G
*Kamundai*	*D. kutejensis*	.	.	T	C	.
*Lai Lidung*	*D. kutejensis*	.	C	T	C	.
*Pampaken*	*D. kutejensis*	.	C	T	C	G
*Pampaken Burung Kecil*	*D. kutejensis*	.	C	T	C	G
*Durian Daun*	*D. lowianus*	G	C	T	C	.
*Durian Malutu*	*D. lowianus*	.	C	T	C	G
*Lahung Alang*	*D. lowianus*	.	C	T	C	G
*Durian Burung Besar*	*D. excelsus*	.	.	.	.	.
*Mantuala Batu Hayam*	*D. excelsus*	.	.	T	C	.
*Maharawin Hamak*	*D. oxleyanus*	.	C	T	C	-
*Karantungan Besar*	*D. oxleyanus*	.	C	T	C	G
*Durian Burung*	*D. acutifolius*	.	.	.	.	.
*Lahung*	*D. dulcis*	.	C	T	C	.
*Durian Kura-Kura*	*D. testudinarium*	.	C	T	C	G
Indian Kapok*	*Bombax ceiba*	.	.	T	C	.
Consensus		A	A	G	G	C

*An outgroup; ^a^substitution-transition; ^b^substitution-transversion

**Table 4:** The AMOVA for durian (*Durio* spp.) germplasm, both inter- and intra-species levels

Source of variation	Sum of square	Variance components	Percentage variation	Fixation indices
Among populations (inter-species)	1923.89	9.75	5.62	*F*_IS_ = 1.00000
Among individuals within populations (intra-species)	3931.00	163.79	94.38	*F*_ST_ = 0.05618
Within individuals	0.00	0.00	0.00	*F*_IT_ = 1.00000
Total	5854.89	173.54	100.00	

### Genetic relationship and divergence

**Fig. 4:**
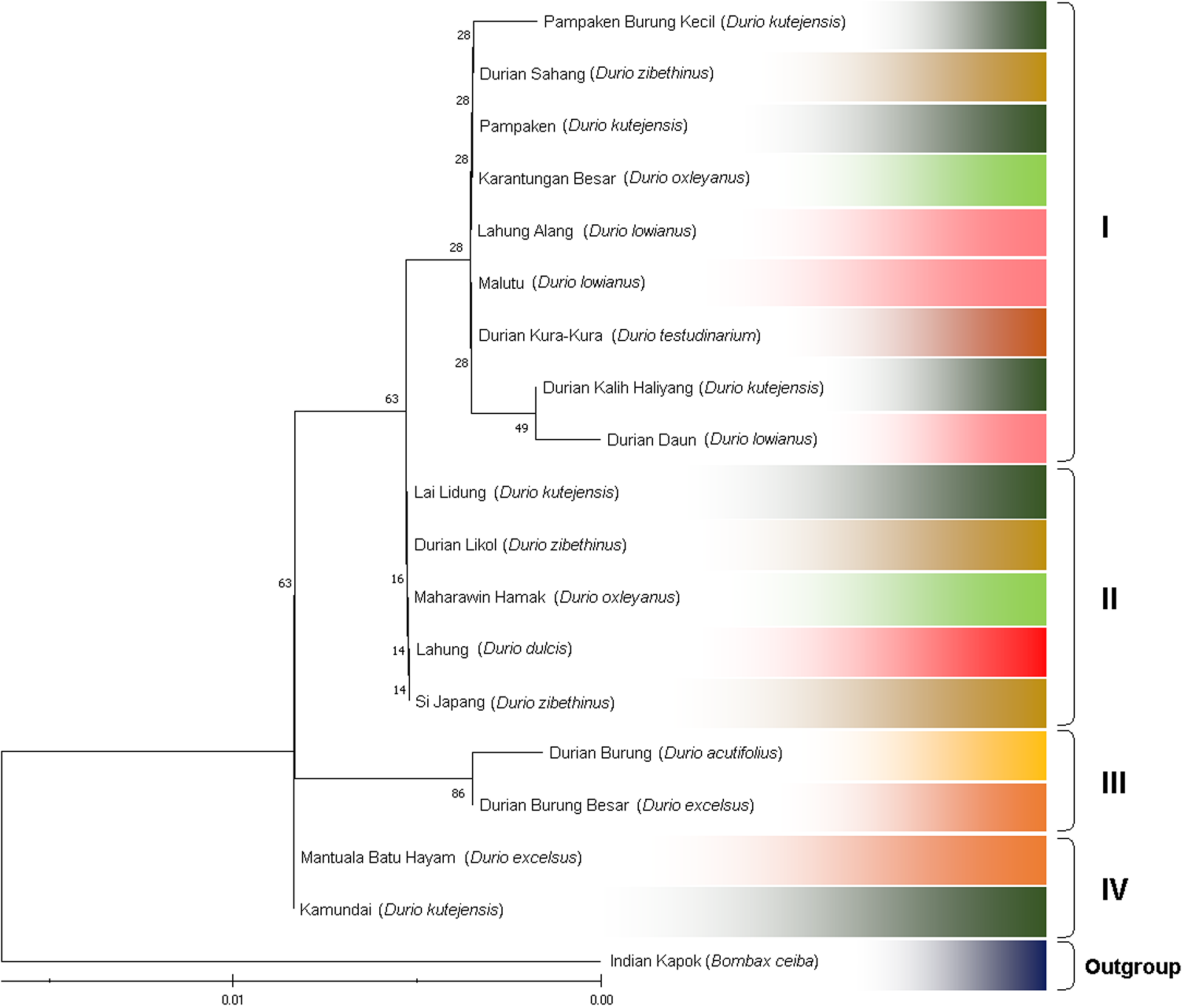
Genetic relationship of exotic durian (*Durio* spp.) germplasm revealed by maximum likelihood (ML) and bootstrap analyses for 1000 replicates

**Fig. 5:**
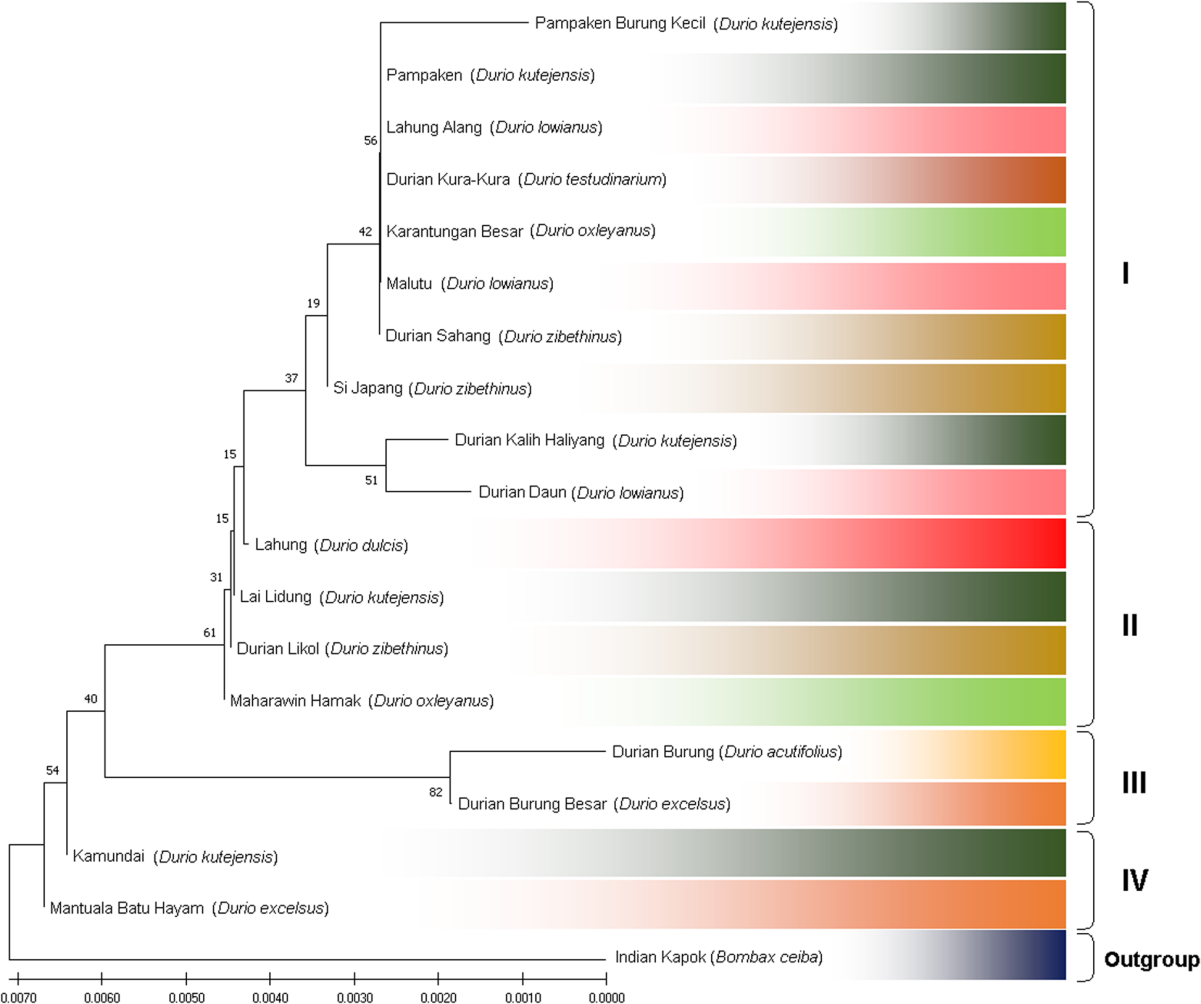
Genetic relationship of exotic durian (*Durio* spp.) germplasm revealed by Neighbor-Joining (NJ) and bootstrap analyses for 1000 replicates

The durian (*Durio* spp.) germplasm from Kalimantan, Indonesia, shows unique relationships. This uniqueness lies in the number and composition of durian members in each clade formed. In general, following the maximum likelihood (ML) and neighbor-joining (NJ) methods, this germplasm is grouped into four main clades (Figs. 4 and 5, respectively). In this case, the first clade (I) is the largest, composed of nine durian germplasm for ML and ten for NJ. Clade II is the next largest consisting of five durian samples for ML and four for NJ. Clades III and IV consisted of two individuals, both ML and NJ. Interestingly, most of the durian samples were consistent in the same clade, both for ML and NJ, except for *Durian Si Japang* (*D. zibethinus*), which belongs to clade II in ML and clade I in NJ (Table 5).

**Table 5:** Grouping of durian (*Durio* spp.) germplasm based on ML and NJ methods

Local name	Species	Clade	
		ML	NJ
*Durian Sahang*	*D. zibethinus*	I	I
*Kalih Haliyang*	*D. kutejensis*	I	I
*Pampaken*	*D. kutejensis*	I	I
*Pampaken Burung Kecil*	*D. kutejensis*	I	I
*Durian Daun*	*D. lowianus*	I	I
*Durian Malutu*	*D. lowianus*	I	I
*Lahung Alang*	*D. lowianus*	I	I
*Karantungan Besar*	*D. oxleyanus*	I	I
*Durian Kura-Kura*	*D. testudinarium*	I	I
*Durian Si Japang*^a^	*D. zibethinus*	II	I
*Durian Likol*	*D. zibethinus*	II	II
*Lai Lidung*	*D. kutejensis*	II	II
*Lahung*	*D. dulcis*	II	II
*Maharawin Hamak*	*D. oxleyanus*	II	II
*Durian Burung*	*D. acutifolius*	III	III
*Durian Burung Besar*	*D. excelsus*	III	III
*Kamundai*	*D. kutejensis*	IV	IV
*Mantuala Batu Hayam*	*D. excelsus*	IV	IV
*Indian Kapok*	*Bombax ceiba*	Outgroup	Outgroup

^a^Inconsistent in grouping

**Fig. 6:**
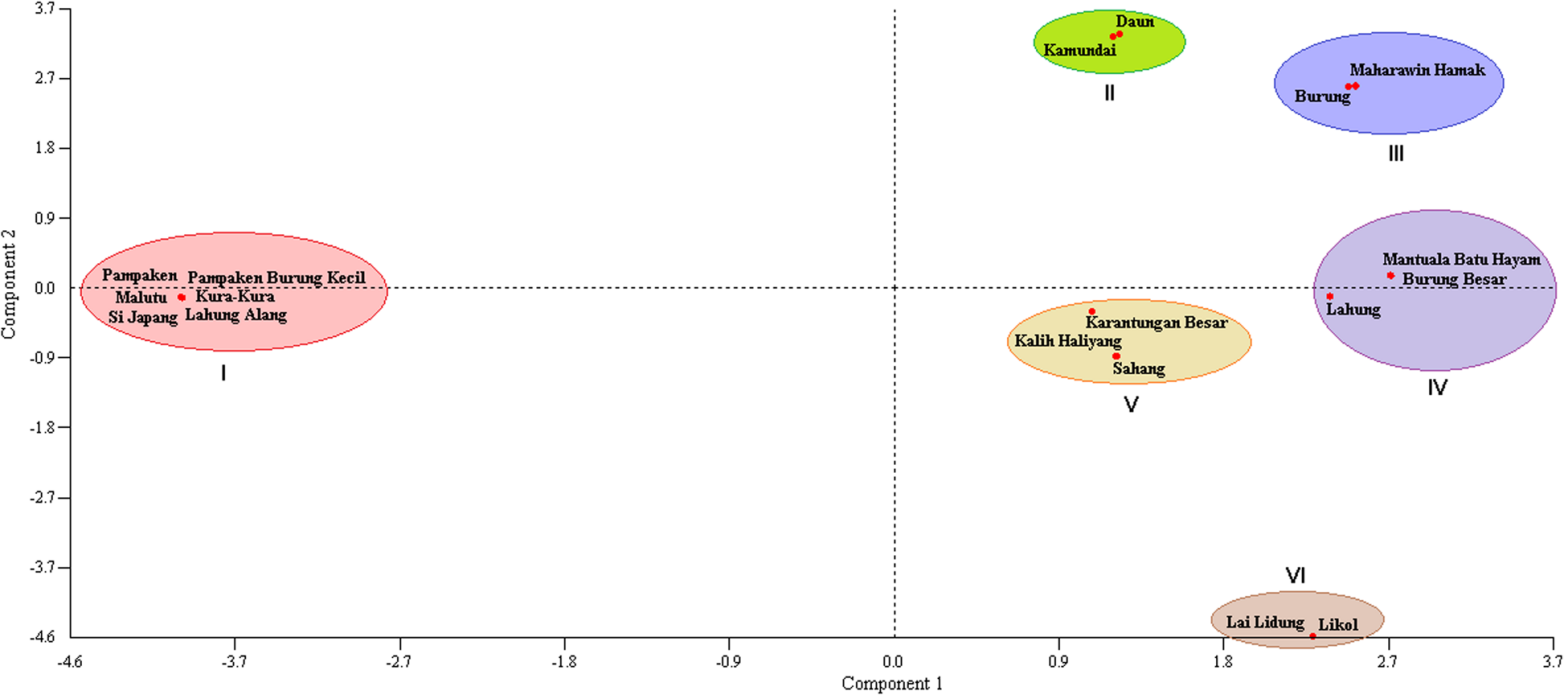
Grouping of exotic durian (*Durio* spp.) germplasm revealed by principal component analysis (PCA) method

The PCA has generated differences in germplasm grouping. In this case, the durians have separated into six groups (Fig. 6), where group I was a largest and compose by six durians, i.e., *Pampaken*, *Pampaken Burung Kecil*, *Malutu*, *Kura-Kura*, *Lahung Alang*, and *Si Japang*. Meanwhile, other groups have consisted of two (IV and V) and three (II, III, and VI) members only (Fig. 6).

**Fig. 7:**
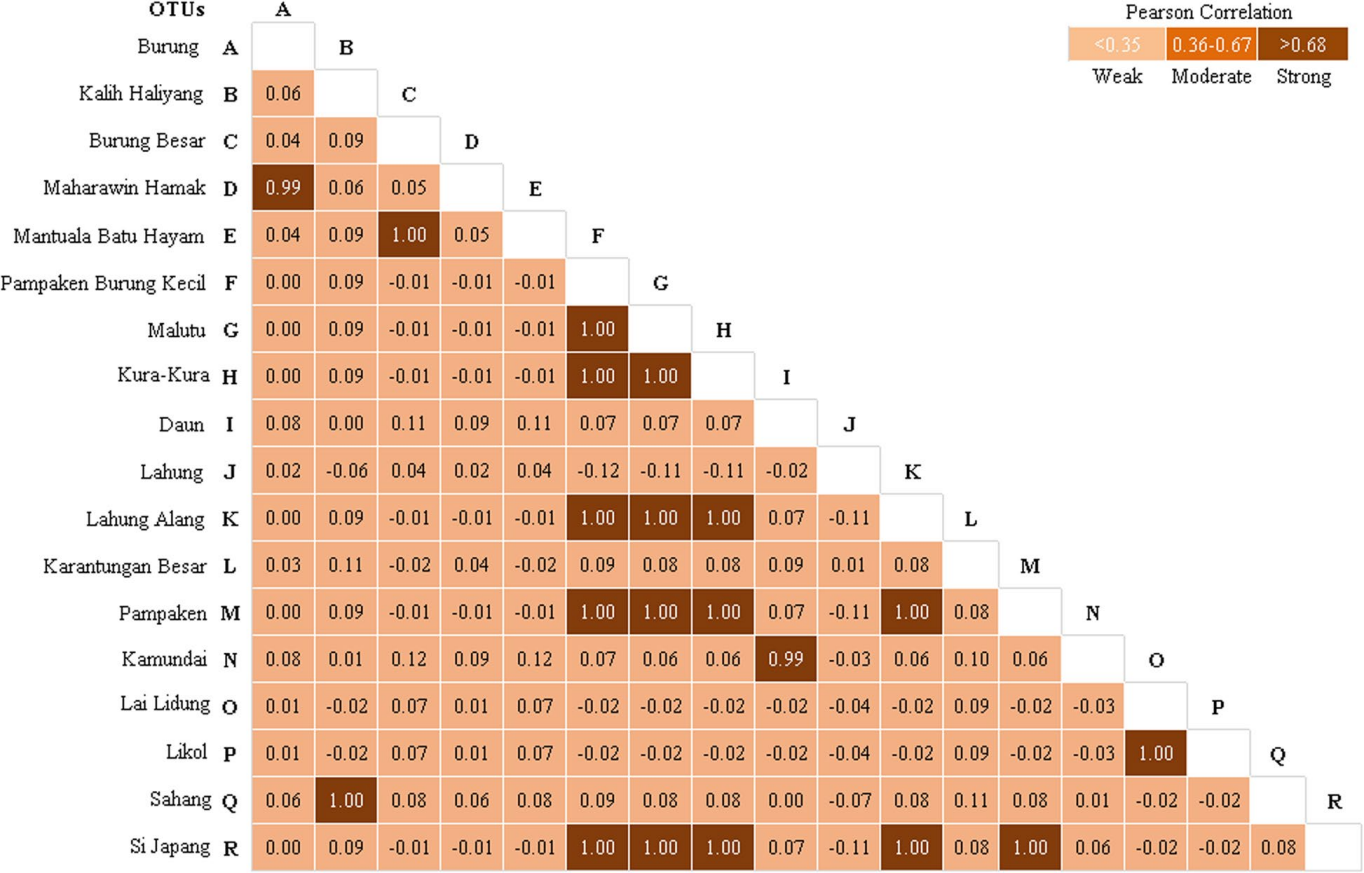
Pearson correlation coefficient among exotic durian (*Durio* spp.) germplasm based on the *rbc*L region

The divergence analysis (Table 6) showed that by species group, *D. zibethinus* was very closely related to *D. exleyanus*. Meanwhile, the farthest shows by *D. lowianus* and *D. excelsus*. Overall, the divergence analysis (Table 7) show a relationship between all samples (individuals), where 29 durian pairs are very closely related, and the farthest shown by *Durian Burung* (*D. acutifolius*) and *Kalih Haliyang* (*D. kutejensis*), and *Pampaken Burung Kecil* (*D. kutejensis*) with *Durian Burung* (*D. acutifolius*) as well, at a divergence coefficient of 0.011. The Pearson correlation analysis confirms that twenty pairs of individual durians have a strong relation (Fig. 7), for example, between *Maharawin Hamak* and *Durian Burung* as well as *Mantuala Batu Hayam* and *Durian Burung Besar* (Fig. 7).

**Table 6:**
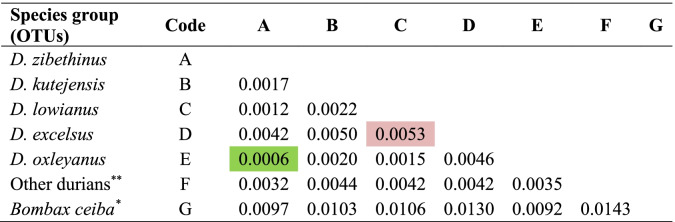
Genetic divergence among durian species (*Durio* spp.) used in this study

^a^An outgroup; ^b^including *D. acutifolius*, *D dulcis*, and *D. testudinarium*; green highlight = closest related; red highlight = farthest related

**Table 7:**
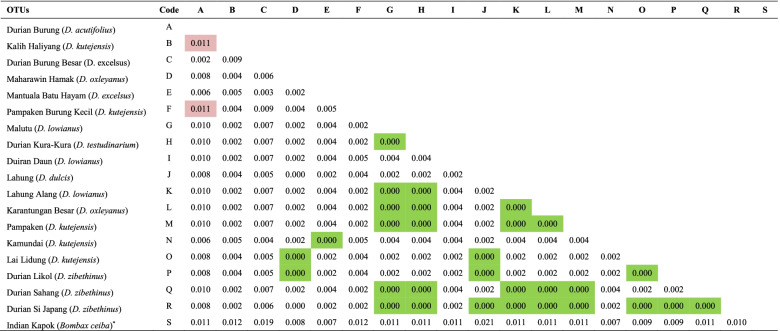
Genetic divergence among all exotic durian (*Durio* spp.) germplasm used in this study

^a^An outgroup; green highlight = closest related; red highlight = farthest related

## Discussion

### The *rbcL* region of the durian

The ribulose-1, 5-bisphosphate carboxylase/oxygenase, or *rbc*L, is a functional gene in the chloroplast genome engaged primarily in plant photosynthesis [32]. This gene is found in the chloroplast genome’s large single-copy (LSC) region and exhibits high similarity across plant germplasm [33]. According to Singh and Banerjee [34], this gene has a 600–800 nucleotide intergenic spacer. The *rbc*L gene contains around 1400 nucleotides that code for the large subunit protein, and the length varies significantly among flowering plants or Angiosperm [35].

In this study, the *rbc*L region of durians has different lengths, ranging from 527 to 578 bp (Table 2). These differences, both partial and complete, have been reported by several researchers. For example, Kumekawa et al. [36] have reported that durian (*D. zibethinus*) has a partial *rbc*L of 250 bp, and Amandita et al. [37] about 500 bp. In complete, this germplasm has the *rbc*L sequence of 1428 bp [38].

Further, a new DNA barcoding motif was discovered in the multiple sequence alignment of the *rbc*L of durians, in which a conserved region is introduced by polymorphism or other mutational events (Fig. 3). Based on Table 2, the *rbc*L durians of Kalimantan showed 23 variable sites or mutational events, and all are substitutions (transition-transversion), and no indels are present. According to Clegg [39], complete codon insertions/deletions are occasionally found in the gene, demonstrating a conservative pattern of nucleotide replacement. In general, grasses and other plant species such as Orchidales, Liliales, Bromeliales, and Arecales have a >5-fold differential in *rbc*L substitution rate [39].

According to Dong et al. [40], this gene represents distinctions in molecular evolution mode and tempo in angiosperms, monocotyledons, Gramineae, and Elymus. In another study, the inter/intrageneric levels of *rbc*L were highly efficient in Cornaceae, Cupressaceae, Ericaceae, and Graniaceae [34]. The *rbc*L gene evolved more quickly in annual plants, particularly in the Asteridae and Poaceae families, and was dubbed “most morphologically advanced forms” in these families [41].

### Genetic diversity and its benefits

In this study, exotic durian (*Durio* spp.) germplasm originally from Kalimantan, Indonesia, has a low genetic diversity, shown by nucleotide diversity (π%) of 0.24 (Table 2). The low level of genetic diversity may be attributed to a combination of founder effects and subsequent bottlenecks encountered in its short domesticated history [42]. While the founder effect is a ubiquitous domestication bottleneck, millennia of cultivation and dissemination into new habitats have provided a considerable opportunity in selecting novel diversity in most crops [42].

Referred to Teixeira and Huber [43], low genetic diversity is often interpreted as an indicator of inbreeding depression and increased genetic drift. In other words, inbreeding, genetic drift, restricted gene flow, and small population size contribute to a genetic diversity reduction. Accordingly, populations lacking genetic diversity often exhibit an increased extinction rate [44]. Ujvari et al. [45] also reported that a decline in genetic diversity is linked to an increased risk of inbreeding depression, resulting in decreased growth rate, fertility, fecundity, and offspring viability, as well as in increased vulnerability to pathogens. Furthermore, a loss of genetic diversity would harm individual fitness with increased susceptibility to disease and parasites [44] and limits a population’s ability to respond to threats in reduced long- and short-term survival of endangered species [46].

Compared to other studies with similar markers used, durian (*Durio* spp.) germplasm from this region has a high diversity. For example, tidal swamp rice (*Oryza sativa*) shows a genetic diversity of 0.086. According to Teixeira and Huber [43], high levels of genetic diversity are beneficial to promoting population survival and guaranteeing the adaptive potential of natural populations in the face of rapidly changing environmental pressures. These principles are reflected in strategies such as genetic rescue, where the genetic diversity of a threatened or endangered population is increased by facilitating gene flow from a population with high levels of diversity [43].

However, emerging genetic diversity strongly correlated with the polymorphic or mutation found in a target region. According to Frankham et al. [47], genetic diversity and mutational events are two things that are related. In this study, the *rbc*L region of the durian germplasm has generated 23 variable sites with a transition/transversion (Ti/Tv) bias value of 1.00 (Table 2). Multiple alignments revealed that transversion is more than transition (Fig. 3 and Table 3). Guo et al. [48] have reported that the first mutation is a more frequent encounter in this sequence and has higher regulatory effects than transitions. However, a pattern of the last mutation is favored several times over transversions is commonly occur in molecular evolution [49, 50].

Regardless of the presence of mutations in the *rbc*L sequence of durians, genetic diversity is essential for plant genetic resources conservation, breeding practices, and preventing genetic basis erosion of breeding populations [14]. For these purposes, examining genetic diversity is essential in managing threatened species or taxa [46]. According to Teixeira and Huber [43], conservation genetic practice rests on the assumption that measured levels of diversity provide a direct indicator of the degree to which genetic factors contribute to the risk of extinction. For crop improvement, genetic diversity is beneficial for parental selection [51] or selecting parents with genetically divergent [14]. In this context, determining populations with a high level of genetic diversity will become a valuable resource for broadening the genetic base or gene pool of germplasm, as this enables the identification of superior alleles for several traits [51].

Following the AMOVA (Table 4), the durian germplasm has a higher variation (94.38%) at the intra-species level than the inter-species one (5.62%). It means that the future durian breeding program can be oriented to outcrossing, as was done by Hariyati et al. [10] and Prihatini et al. [9]. According to Uji [6], several wild durian species, except *D. zibethinus*, have potential genes that can be incorporated into this program, such as being resistant to diseases and having a high tolerance for environmental challenges.

### Genetic relationship and divergence

The phylogenetic study or genetic relationships is also beneficial for plant genetic conservation and breeding practices [14]. For the first program, this study can be applied in inferring species and their evolutionary history, including species delimitation, genetic differentiation, and gene flow [52]. In other words, this information is given the objective metrics for conservation purposes in the past evolution history, genetic status of species in the present time, and management program for future ones [52]. For the second or last purposes, information of this relationship is usable in predicting the genetic diversity of the offspring when individuals or populations cross [5].

In this study, the durian (*Durio* spp.) germplasm from Kalimantan, Indonesia, shows unique relationships, mainly based on the number and composition of durian members in each clade or group formed. In general, following the maximum likelihood (ML) and neighbor-joining (NJ) methods, this germplasm is grouped into four main clades (Figs. 4 and 5, respectively). According to the PCA, this germplasm was separated into six groups (Fig. 6). Interestingly, both for ML and NJ, most of the durian samples were consistent in the same clade, except for *Durian Si Japang* (*D. zibethinus*), which belongs to Clade II in ML and Clade I in NJ (Table 5). Briefly, these phylogenetic trees (Figs. 4 and 5) and grouping illustrated the closeness and distant relationship between the samples.

The divergence analysis (Table 6) showed that by species group, *D. zibethinus* was very closely related to *D. exleyanus*. Meanwhile, the farthest shows by *D. lowianus* and *D. excelsus*. By ITS and *ndh*F markers, Nyffeler and Baum [53, 54] reported a close relationship between *D. zibethinus* and *D. oxleyanus*. Such relationships were also stated by Santoso et al. [55] using RFLP, Santoso et al. [12] by microsatellite, and Santoso et al. [56] with ITS.

However, within individuals (Table 7), 29 durian pairs are very closely related, and the farthest shown by *Durian Burung* (*D. acutifolius*) and *Kalih Haliyang* (*D. kutejensis*), and *Pampaken Burung Kecil* (*D. kutejensis*) with *Durian Burung* (*D. acutifolius*) as well, at a divergence coefficient of 0.011. Following the Pearson correlation analysis, only 20 pairs of individual durians have a strong relation, for example, *Maharawin Hamak* and *Durian Burung* as well as *Mantuala Batu Hayam* and *Durian Burung Besar* (Fig. 7). According to Acquaah [5], crossing individuals with distant relationships may generate high genetic diversity in the offspring. Conversely, crossing individuals with very close related may result in offspring with a low or narrow genetic diversity. In general, crossing individuals with a very close relationship is tends to avoid, as inbreeding occurs in the offspring [57]. Thus, our results are essential in supporting the future durian genetic conservation and breeding practices.

## Conclusion

Following the *rbc*L region, the exotic durian (*Durio* spp.) germplasm originally from Kalimantan, Indonesia, has a low genetic diversity (π%=0.24). However, following the phylogenetic and principal component analyses, this germplasm is separated into four main clades and six groups, respectively. In this case, *D. zibethinus* is very closely related to *D. exleyanus*. Meanwhile, *D. lowianus* and *D. excelsus are* the farthest. Individually, 29 durians were very closely related, and the was farthest shown by *Durian Burung* (*D. acutifolius*) and *Kalih Haliyang* (*D. kutejensis*) as well as also *Pampaken Burung Kecil* (*D. kutejensis*) and *Durian Burung* (*D. acutifolius*) with a divergence coefficient of 0.011. The Pearson correlation analysis confirms that 20 pairs of individual durians have a strong relation, shown by (e.g.) *Maharawin Hamak* and *Durian Burung*, also *Mantuala Batu Hayam* and *Durian Burung Besar.* Our results may provide a fundamental paradigm in supporting the durian genetic conservation and breeding practices in the future, locally and globally.

## Data Availability

The datasets used and analyzed in the present study are presented in the article, and the *rbc*L sequences of this germplasm were deposited in GenBank with accession numbers.

## Abbreviations

ITS: Internal transcribed spacer
ML: Maximum likelihood
NJ: Neighbor-joining
PCA: Principal component analysis
RFLP: Restriction fragment length polymorphism
